# Biochemical efficacy and safety trial of vitamin D (BEST-D): finding an appropriate dose to test in a large randomized trial

**DOI:** 10.1186/1745-6215-14-S1-P45

**Published:** 2013-11-29

**Authors:** Jane Armitage, Harold Hin, Joseph Tomson, Mike Lay, Mike Hill, Robert Clarke

**Affiliations:** 1 University of Oxford, Oxford, UK; 2 Hightown Surgery, Banbury, UK

## 

Understanding the shape and strength of risk factor associations with disease in observational studies is essential for the successful design of risk factor-modification trials. Observational studies show that low plasma levels of 25-hydroxy vitamin D (25[OH]D), a blood marker of vitamin D status, are linearly related with bone mineral density and fractures, in addition to higher risks of vascular and non-vascular mortality. Over the last 20 years, several trials of vitamin D3 have assessed the effects of supplementation with equivalent daily doses of 400-800 IU on risk of fracture, but results have been conflicting. A meta-analysis of such trials reported a 14% (95%CI 4-23%) reduction in non-vertebral fractures, but the results suggest that plasma levels of 25[OH]D need to be at least 80-90 nmol/L to provide fracture protection. Moreover, previous trials also indicate an average increase of only about 7-10 nmol/L in plasma 25[OH]D for each 400 IU of vitamin D3 given daily. Consequently, at least 2000 IU daily may be required to increase plasma levels of 25(OH)D from an average winter level of 30 nmol/L (typical in the UK) to 90 nmol/L, suggesting that the previous trials have used insufficient doses of vitamin D3 to detect the epidemiologically predicted differences in clinical outcomes. To help determine the optimum dose of vitamin D3 for prevention of fractures and other health outcomes, we are comparing the biochemical efficacy and safety of 2000 and 4000 IU daily (versus placebo) vitamin D3 in a pilot study of 305 healthy people aged >65 years.

